# How does the West African Health Organisation (WAHO) contribute to the evidence based decision-making and practice during COVID-19 pandemic in ECOWAS region?

**DOI:** 10.11604/pamj.supp.2020.37.1.25625

**Published:** 2020-10-02

**Authors:** Issiaka Sombié, Ermel Johnson, Virgil Lokossou, Tete Amouh, Abdourahmane Sow, Nanlop Ogbureke, Stanley Okolo

**Affiliations:** 1 West African Health Organisation, 175, Avenue Ouezzin Coulibaly, 01BP: 153 Bobo Dioulasso 01, Burkina Faso

**Keywords:** Evidence use, decision making, practice, COVID-19, West Africa

## Abstract

The COVID-19 pandemic required policy makers to make urgent decisions to limit the spread of the disease. International and regional health bodies and research institutions have a role in supporting decision makers and health actors in providing accurate and timely research evidence and guidance in decision making and practice. In ECOWAS region, the West African Health Organisation (WAHO) has experience in promoting evidence use decision making and practice as part of its role as Health Policy and Research Organisation. Promoting the use of evidence to influence policy and practice is possible through various approaches including training, the development of guides and policy briefs, the synthesis and sharing of evidence, and the organisation of meetings to share experiences. In the context of the COVID-19 pandemic, WAHO has deployed several approaches to bring the use of evidence to decision-makers and stakeholders to influence policy and practice. To improve practices, WAHO has organized regional training workshops on laboratory diagnostic, surveillance and simulation exercises of outbreak response for key actors, as well as webinars on different aspects of COVID-19 pandemic surveillance, coordination and management. In addition, a synthesis of the most recent evidence and epidemiologic models were developed to enlighten decision makers in selecting and implementation response interventions.

## Commentary

COVID-19, a disease discovered in China in December 2019, was declared a public health emergency of international concern in January 2020 and a pandemic in March 2020 [1] by the World Health Organisation (WHO). COVID-19 pandemic required policy makers to make urgent decisions to limit the spread of the disease. These decisions covered several areas of disease control including governance, surveillance, contact tracing, isolation, patient management and non-pharmaceutical measures to help limit the spread of the disease. Knowledge and recommendations to contain and stem the spread of the pandemic are uncertain and changing over time. As a result, decision-makers have turned to international or regional health institutions, health research centres and institutes for guidance and direction in their decision-making processes. In this commentary, we report on the experience of the West African Health Organization (WAHO), the specialised health agency of the Economic Community of West African States (ECOWAS).

### WAHO past experience in promoting the use of evidence in decision-making and practice

The first objective of WAHO in his protocol of creation is to promote research on the main endemic diseases in the sub-region and to undertake activities aimed at the control and eradication of these diseases. This objective shows the importance of promoting of the research and use of evidence in WAHO's mission. To be successful in this area, WAHO worked to develop national health research systems by working according the framework of Pang *et al*. with the functions of promoting governance, financing, institutional and individual capacity building, dissemination and use of evidence and promotion of regional collaboration [2]. In the area of promoting the use of evidence, the institution currently plays the role of research policy organization for health research within the framework of the Canadian Initiative on Innovation for Maternal and Child Health in Africa (IMCHA) [3]. In this initiative, it plays the role of bringing researchers and decision-makers together to better use evidence to influence policy and practice. A situation analysis conducted allows a better understanding of the process of evidence use in policymaking and practice [4]. This analysis leads to a regional knowledge transfer framework to close the gap of research evidence utilisation in decision-making and practice. Thus, WAHO is currently promoting innovative approaches to facilitate the use of evidence through capacity building of individual researchers and policy makers [5], the establishment of platforms for exchange between researchers and decision-makers [6], and the development of regional guidelines for evidence-based policy formulation [7]. The commitment of high-level health decision-makers in the sub-region resulted in the adoption of a resolution by the Ministers of Health of the region on the importance of using evidence [8].

### WAHO approach to support evidence use in COVID-19 pandemic response

Promoting the use of evidence to influence policy and practice is possible through various approaches including training, the development of guides and policy briefs, the synthesis and sharing of evidence, and the organisation of meetings to share experiences [9]. In the context of the COVID-19 pandemic and based in his past experience, the institution has deployed several approaches to bring the use of evidence to decision-makers and stakeholders to influence policy and practice.

**Capacity building for evidence-based practices:** the knowledge of health actors and the public on the evolution of the epidemic in the sub-region is regularly updated. Indeed, WAHO has developed a dynamic platform for daily updates on epidemiological information on the fifteen ECOWAS countries [10]. In addition, press conferences are organised as needed to enlighten public opinion. To improve practices, WAHO has organised a regional training workshop in February before any COVID-19 notification in the region that has enabled laboratory personnel from the 15 countries to acquire the knowledge and practical skills that have enabled them to return to the countries to enhance their diagnostic practices. The training has enabled all countries to be able to perform diagnostic PCR tests in their countries and improve the availability of COVID-19 laboratory testing site from two before the epidemic to one hundred and fifty actually. In addition, WAHO developed laboratory technical guidelines for SARS-CoV-2 sample management and diagnostic. To avoid in stock out of laboratory reagents in the region, a regional platform was set up to collect and monitor weekly the number of tests performed, the availability of diagnostic kits and others laboratory consumables. In addition, a laboratory based short model-built helps identify the needs of diagnostic kits at monthly basis and supply countries. Then, to enable those in charge of the response coordination to be equipped with tools on the organization of the response, a workshop with table top simulation exercises in early march 2020 helped familiarize four persons per country with the fight against COVID before the arrival of the disease in different countries. Continuing to improve practices and update knowledge, WAHO has organised 35 webinars for the benefit of 3367 health workers involved in the fight against COVID. The webinar's topics included infection prevention and control, the epidemiology of COVID and its implications for prevention and surveillance, contact tracing, fundamentals of COVID-19 patient management, steps in COVID-19 laboratory diagnosis, psychosocial impact on caregivers and patients and mitigation approaches, cross-border strategies to address COVID-19 and risk communication. These webinars allowed the dissemination of international and regional guides and exchanges of experiences between actors.

**Influence of policies and other measures by evidence:** first, WAHO has produced evidence synthesis made available to the Ministers of Health in certain areas including the use of chloroquine, hydroxychloroquine and azythromycin in the management of patients with COVID-19. It has also shared information on the state of research in the field of drugs and vaccines at technical meeting of the Ministers of Health on the follow-up of the recommendations of the ECOWAS Heads of State. Second, to help maintain essential healthcare services during the epidemic, WAHO with partners including WHO Afro, JPhiego, IntraHealth conducted a situation analysis with programme managers to identify challenges and potential solutions to improve essential health services during the COVID-19 pandemic. A webinar organised following the survey to present the findings and guides needed to support the maintenance of maternal and child health services. Thirdly, WAHO has developed epidemiological models to help improve the response by monitoring the evolution of reproductive rate and estimating the needs in terms of testing kits, protection and care materials. This information has helped WAHO management to tailor its support to the actual needs of countries. These models were presented to countries national coordination institute, and made available on a dynamic electronic platform. Fourthly it organized weekly regional online regional meetings with all countries to share innovative practical experiences in responding to each other's needs. Finally, WAHO support some regional institutions in developing and testing the performance of rapid diagnostic test to facilitate their adoption by the countries in their testing strategies.

**Future directions:** WAHO is currently planning the establishment of communities of practice that will include decision-makers, researchers, practitioners and partners to continue the exchange between stakeholders for the use of best evidence in strategic and programmatic decision-making. Furthermore, WAHO is developing risk awareness, information, and education and communication tools to help improve risk communication to improve the health literacy of populations. It will also organize the Good Practice Forum in 2021, which will promote the innovations and best practices identified during the current pandemic response. Finally, it is planned to set up a network of champions in countries for advocacy, training of knowledge brokers and various actors who will contribute to the identification, dissemination and support for the use of evidence.

## Conclusion

This commentary highlights the facilitation role that a regional health institution can play in empowering country actors and promoting evidence to influence decisions and response practices. This role of facilitating the use of evidence will be maintained as a key activity of the ECOWAS Regional Centre for Disease Surveillance and Control, the ECOWAS regional framework to support countries in epidemic preparedness and response.
